# A Serious Charge

**Published:** 1912-06

**Authors:** R. H. L. Bibb


A SERIOUS CHARGE.—I extract the following incredible story from the (New York) *Medical Record* of May 4, 1912:

"From San Antonio, Texas, comes the report of a mild epidemic of cerebro-spinal meningitis with 26 deaths in 57 cases. An observer of the conditions, the wife of an army officer, protesting against the lack of proper quarantine regulations, states that most of the cases have originated in the schools which, nevertheless, are allowed to remain in session, that the Board of Health does not allow cases of the disease to be reported, and that little is done in the way of prevention, the excuse being that publicity might cause a panic. The city is the great center for the tourist travel of the Southwest, and the writer thinks that active measures should speedily be taken to protect those who are now kept in ignorance. The army post has been placed under effective quarantine, but the townspeople are apparently unaware of the disease."

It is impossible for one who, like the writer of this, knows the professional and social standing of the excellent gentlemen who compose the San Antonio Board of Health to believe they would perpetrate so unpardonable an outrage upon an innocent public, at any time and under any circumstances, and more especially at this time, when so many people from all sections of the country are attending the various important conventions being held in that historical city, even though the general knowledge that there was "a mild epidemic of cerebro-spinal meningitis" in San Antonio would keep thousands away who would otherwise attend.

The report, coming as it does, in the columns of a medical publication of the enviable and deservedly high standing which the *Record* enjoys, will attract attention the world over, and will do the city of the Alamo an immense amount of harm from every standpoint, and it is now incumbent upon the city's health authorities to show to the world that there is not, and that there *has not* been, any foundation for the charges which the *Record* makes.

Should it turn out, as it probably will, that the *Record* has been victimized, and should the San Antonio Health Board need the services of such an institution, it has been suggested that Dr. Daniel's Mendacitorium, which, like our penitentiaries, asylums, hospitals and jails, is always filled to overflowing, may possibly find room for a patient or two more.

R. H. L. Bibb.

